# Do higher dialysate calcium concentrations increase vascular stiffness in haemodialysis patients as measured by aortic pulse wave velocity?

**DOI:** 10.1186/1471-2369-14-189

**Published:** 2013-09-08

**Authors:** Evangelia Charitaki, Andrew Davenport

**Affiliations:** 1 UCL Centre for Nephrology, Royal Free hospital, University College London Medical School, Rowland Hill Street, London NW3 2PF, UK

**Keywords:** Hypertension, Haemodialysis, Dialysate calcium, Pulse wave velocity, Vascular calcification

## Abstract

**Background:**

Haemodialysis patients have an increased prevalence of hypertension and risk of cardiovascular mortality and stroke. Higher dialysate calcium concentrations have been reported to cause both an acute and chronic increase in arterial stiffness. We therefore looked at changes in arterial stiffness in established haemodialysis patients to determine whether there was a threshold effect of dialysate calcium concentration linked to change in arterial stiffness.

**Methods:**

We performed pulse wave velocity measurements six months apart in patients dialysing with calcium concentrations of 1.0, 1.25, 1.35 and ≥1.5 mmol/l.

**Results:**

289 patients, 62.2% male, mean age 65.5 ± 15.7 years, weight body mass index 25.8 ± 5.4 kg/m^2^ ,47.9% diabetic were studied. Systolic blood pressure (SBP) was 148.4 ± 28.6 mmHg and diastolic blood pressure (DBP) 80.2 ± 15.5 mmHg. Mean pulse wave velocity increased over time (9.66 ± 2.0 vs 10.13 ± 2.16 m/s; p < 0.001), but there was no change in aortic augmentation index (38.7 ± 16.3 vs 39.8 ± 15.6%) or central aortic pressure (149.6 ± 33.3 vs 150.4 ± 31.9 mmHg).

Pulse wave velocity did not differ between the four groups either at start or end of the study, but increased both in the groups dialysing with a calcium concentration of 1.0 mmol/l (9.64 ± 1.94 vs 10.45 ± 1.98 m/s, p = 0.0028) and also with 1.35 mmol/l (9.75 ± 1.96 vs 10.21 ± 2.18 m/s, p = 0.02).

**Conclusions:**

Pulse wave velocity increased over the six months study. As pulse wave velocity increased in the group dialysing using the lowest dialysate calcium, it is likely that factors, other than simple net calcium influx and efflux during dialysis according to dialysate calcium concentration are involved with vascular stiffening.

## Background

Although haemodialysis is an established outpatient treatment for patients with chronic kidney disease, mortality, in particular cardiovascular mortality, remains high. Whereas atheromatous coronary artery disease is the predominant cardiovascular risk factor for the general population, arterio-sclerosis is more commonly found in the haemodialysis patient, leading to increased risk of sudden cardiac arrhythmic death, heart failure and stroke [1].

Vascular calcification, in particular medial calcification, is more common in haemodialysis patients, and although the pathogenesis of soft tissue calcification is multifactorial, it has been suggested that repeated episodes of hypercalcaemia can increase the risk of vascular calcification [2].

Calcification of major arteries increases arterial stiffness and pulse wave velocity. Increased aortic stiffness and the associated elevated pulse pressure in central arteries has been shown to be a strong and independent predictor of cardiovascular events in both the general population and also patients with chronic kidney disease [3]. Several small studies have reported that pulse wave velocity can increase following a single haemodialysis treatment when using a higher dialysate calcium concentration compared to a lower concentration [4,5]. Dialysing against higher dialysate calcium concentrations leads to greater prevalence of hypercalcaemia [6], and other small studies have reported that pulse wave velocity increases over time in both haemodialysis [7-9] and peritoneal dialysis patients [10] dialysed using higher dialysate calcium concentrations than lower. Currently there is no consensus on the optimum dialysate calcium concentration [11], and we reviewed pulse wave velocity changes in a cohort of chronic haemodialysis patients dialysed with various calcium concentrations to determine whether there were any discernible changes over time.

## Methods

289 adult patients who had pulse wave velocity (PWV) measurements predialysis 6 months apart and had continued to dialyse with the same dialysate calcium concentration were reviewed. Aortic-brachial pulse wave velocity was measured using the Tensio Clinic Ateriograph (TensioMed Kft., Budapest, Hungary) an applanation oscillometric device which has been validated against direct invasive measurements [12]. The distance between jugular notch and symphysis pubis was measured with either the patient standing upright, or lying flat using a specially designed and adjustable calibrated caliper. Pulse wave velocity measurements were subsequently made in the recumbent position in the non-fistula arm after patients had rested for a minimum of 10 minutes, using the appropriate sized cuff for the patients’s arm . Patients were advised not to take nitrates prior to measurement of pulse wave velocity. PWV measurements were not able to be recorded in patients with atrial fibrillation, other arrhythmias, those with fistulae in both arms, and patients with no recordable upper arm blood pressure recordings. Augmentation indices (AXi) were calculated for the aorta (AoAXi) and brachial arteries (BrAXi) as the difference between the amplitudes of the late (backward) systolic wave (P2) and the early (forward) systolic wave (P1) over the pulse pressure (PP) and multiplied with 100. AXi = (P2-P1)/PP x100, and adjusted for heart rate. Diastolic relaxation area (DRA), a measure of diastolic filling of the left coronary artery was measured and then adjusted for heart rate. Three inflations of the brachial arterial cuff were recorded, and if the pulse wave velocity differed by >0.5 m/s, the reading was repeated, and mean value recorded.

Patients were dialysed thrice weekly, targeted to achieve an on-line clearance of ≥1.4 (Fresenius 4008/5008, Fresenius Bad Homberg, Germany, Dialog+, B Braun Medical Inc, Melsungen, Germany), mean session time 3.96 ± 0.38 hours, using polysulfone dialyzers (Nipro, Osaka, Japan) [13] with ultrapure quality dialysate, and anticoagulated with tinzaparin (Leo Laboratories, Market Harborough, UK) [14]. 77.4% of patients dialysed using an arterio-venous fistula.

Biochemical investigations were performed using standard automated multichannel analyser, with albumin measured by the bromcresol green method (Roche Integra, Roche diagnostics, Lewes, UK). Hypotensive episodes during dialysis requiring changing ultrafiltration rates and intravenous fluid administration were recorded. Standard 2 dimensional echocardiograms were performed on a non-dialysis day (Philips IE33, Philips Medical Systems, Eindhoven, Netherlands).

This retrospective audit complied with the UK NHS guidelines for audit and clinical service development. Measurement of pulse wave velocity was introduced into clinical practice in 2011 and measured as part of an audit of blood pressure control in haemodialysis patients. Echocardiograms were performed as part of transplant assessment.

### Statistical analysis

Statistical analysis used paired students’ t test and Wilcoxon rank sum pair tests for analysis of the whole study cohort (GraphPad Prism version 6.0, San Diego, USA), and by Chi square and one way anova with Tukey post hoc analysis correction, for intergroup analysis (SPSS version 17.0, SPSS Inc, Univ Chicago, Illinois, USA). Data are expressed as mean ± standard deviation, median and inter-quartile range, or percentages. Statistical significance was taken at or below the 5% level.

## Results

Data was available on 289 patients, 62.2% male, mean age 65.5 ± 15.7 years, weight 71.4 ± 16.7 kg, body mass index 25.8 ± 5.4 kg/m^2^ and 47.9% diabetic. 79.5% had a past medical history of hypertension, 29.2% ischaemic heart disease, 19.1% peripheral vascular disease, 14.1% cerebrovascular disease and 5.6% had previous parathyroidectomy. Systolic blood pressure (SBP) 148.4 ± 28.6 mmHg, diastolic blood pressure (DBP) 80.2 ± 15.5 mmHg, with 28.6% prescribed angiotensin converting enzyme inhibitors (ACEIs) or angiotensin receptor blockers (ARBs), 27.2% beta-blockers and 20.9% calcium channel blockers.

Serum albumin was 39.7 ± 3.9 g/l, calcium 2.27 ± 0.17 mmol/l, corrected calcium 2.44 ± 0.16 mmol/l, phosphate 1.48 ± 0.48 mmol/l, alkaline phosphatase 97 (72–135) Iu/l and parathyroid hormone (PTH) 26.8 (13.9-53.5) pmol/l. Median weekly dose of calcitriol prescribed was 2 (0.75-4.5) ug, with 52.9% prescribed calcium containing phosphate binders, 15.6% sevelamer, 13.9% lanthanum carbonate and 7.6% cinacalcet. Serum total cholesterol (TChol) was 3.97 ± 1.1 mmol/l, high density lipoprotein (HDL) 1.27 ± 0.48, low density lipoprotein (LDL) 2.0 ± 0.9 mmol/l and triglycerides 1.3 (0.9-1.9) mmol/l, with 58.2% prescribed HMG CoA 3 reductase inhibitors (statin).

Mean pulse wave velocity increased over time (9.66 ± 2.0 vs 10.13 ± 2.16 m/s ,p < 0.001), but there was no change in aortic augmentation index (38.7 ± 16.3 vs 39.8 ± 15.6%), or central aortic pressure (149.6 ± 33.3 vs 150.4 ± 31.9 mmHg).

As this an audit of clinical practice and not a prospective trial a range of dialysate calcium concentrations were used with different numbers of patients using each of the diaysates, with 18.8% patients using a dialysate calcium concentration of 1.0 mmol/l, 20.9% 1.25 mmol/l, 53% 1.35 mmol/l, 5.9% 1.5 mmol/l and 1.4% 1.75 mmol/l respectively. The groups were well matched for demographics and serum biochemistries (Tables 1 and 2), although more of the cohort dialysing with the lowest calcium dialysate concentration had a history or previous hypertension, higher serum phosphate and parathyroid levels, and were prescribed more oral calcium based phosphate binders and sevelamer hydrochloride, compared to those dialysing against the highest dialysate calcium concentrations.

**Table 1:** Demographics of patients dialysing against different dialysate calcium concentrations

**Dialysate calcium**	**1.0 mmol/l**	**1.25 mmol/l**	**1.35 mmol/l**	**≥1.5 mmol/l**
N	54	60	154	21
age yr	65.9 ± 16.7	64.9 ± 14.5	65.4 ± 15.7	73.4 ± 18.1
male (%)	53.7	65.0	64.9	57.1
weight kg	70.0 ± 15.4	71.6 ± 15.8	72.3 ± 17.9	67.3 ± 12,5
BMI kg/cm^2^	25.7 ± 4.8	26.0 ± 5.5	25.9 ± 5.6	25.4 ± 4.6
diabetic (%)	42.6	43.3	50	57.1
hypertension (%)	94.4*	81.7	74.0	76.2
heart disease (%)	27.8	13.3*	33.8	42.9
TIA/CVA (%)	14.8	13.3	15.6	9.5
PVD (%)	25.9	23.3	16.2	9.8
smoker (%)	7.4	11.7	7.5	9.5
ex-smoker (%)	22.2	23.3	30.6	21.3
AVF (%)	77.8	76.7	70.8	61.9
vintage mo	37.5(21–86)	30(12.3-67.5)	30(12–56)	27(13–56.5)
session h	4.02 ± 0.32*	3.96 ± 0.37	3.96 ± 0.39	3.75 ± 0.42

Results expressed as percentage (%), mean ± SD, or median (interquartile range). Body mass index (BMI), medical history hypertension, ischaemic heart disease (myocardial infarction, coronary artery stenting or bypass surgery), transient ischaemic attack or cerebrovascular accident (TIA/CVA) or peripheral vascular disease (PVD). Arteriovenous fistula (AVF), dialysis vintage in months (vintage mo), dialysis session time (session). Patients dialysing with dialysate calcium 1.5 mmol/l (n = 17) added to those dialysing using calcium 1.75 mmol/l (n = 4) summated due to low numbers. *p < 0.05 by Chi square analysis or anova with post hoc correction vs high calcium dialysate (≥1.5 mmol/l).

**Table 2:** Biochemical data from patients dialysing against different dialysate calcium concentrations

**Dialysate calcium**	**1.0 mmol/l**	**1.25 mmol/l**	**1.35 mmol/l**	**≥ 1.5 mmol/l**
Calcium	2.3 ± 0.17	2.3 ± 0.17	2.25 ± 0.17	2.23 ± 0.16
Calcium corr	2.45 ± 0.17	2.47 ± 0.16	2.43 ± 0.15	2.40 ± 0.13
Phosphate	1.62 ± 0.43*	1.48 ± 0.51*	1.48 ± 0.48*	1.08 ± 0.3
Albumin g/l	39.3 ± 3.3	41.1 ± 3.7*+	39.4 ± 4.2	38.1 ± 3.7
ALP	128(89–180)	87(71–113)	96(69–138)	91(73–108)
PTH	45(22–77)*+	31(15–51)	24(13–52)	16(10–26)
TChol	3.8 ± 1.0	4.0 ± 0.17	4.0 ± 1.0	3.9 ± 1.0
HDL	1.23 ± 0.33	1.28 ± 0.40	1.31 ± 0.55	1.02 ± 0.38
LDL	1.86 ± 0.89	2.09 ± 0.80	2.07 ± 0.94	2.04 ± 0.84
TG	1.40(0.8-1.93)	1.20(0.9-1.1)	1.20(0.9-1.9)	1.65(1.1-2.58)
Statin (%)	48.2	59.3	61	61.9
Calcium	0(0–1.28)*	1.5(0–1.5)	1.0(0–3.0)	1.0(0–3.0)
Sevelamer	0(0–1.0)*	0(0–0)	0(0–0)	0(0–0)
Lanthanum	0(0–250)	0(0–0)	0(0–0)	0(0–0)
Cinacalcet	0(0–30)*	0(0–0)	0(0–0)	0(0–0)
Calcitriol	3(0.94-5.25)	3(0.75-5.25)	1.75(0.75-4.5)	1.5(0.-3)
PTX (%)	5.6	8.3	1.7	0

Data expressed in mmol/l unless otherwise stated. Calcium (measured calcium), calcium corrected for serum albumin (Calcium corr), alkaline phosphatase IU/L (ALP), parathyroid hormone pmol/l (PTH), total cholesterol (TChol), high density lipoprotein (HDL), low density lipoprotein (LDL), triglycerides (TG). Drug prescription: HMG CoA3reductase (statin), calcium carbonate (Calcium) g/day, sevelamer g/day, lanthanum carbonate (lanthanum) g/day, calcitriol (ug/wk), and cinacalcet mg/day, percentage patients with parathyroidectomy (PTX). Results expressed as percentage (%), mean ± SD, or median (interquartile range). Patients dialysing with dialysate calcium 1.5 mmol/l (n = 17) added to those dialysing using calcium 1.75 mmol/l (n = 4) summated due to low numbers. *p < 0.05 by Chi square analysis or anova with post hoc correction vs high dialysate calcium (≥1.5 mmol/l) and + p < 0.05 vs calcium dialysate 1.35 mmol/l.

**Figure 1:**
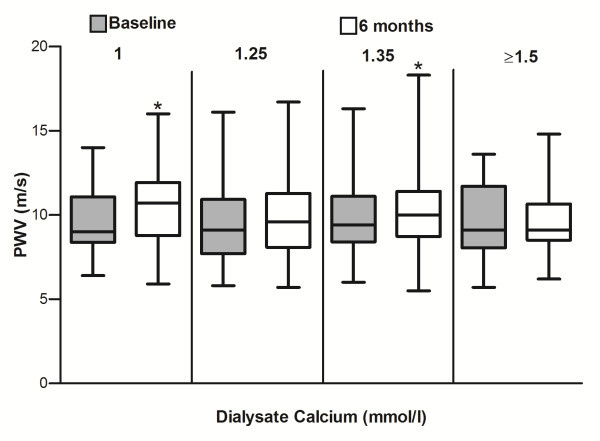
**Pulse wave velocity measured 6 months apart according to dialysate calcium (mmol/l) concentration.** * p < 0.05 compared to baseline reading, with post hoc correction.

Peripheral and central blood pressure measurements were not different between the calcium dialysate cohorts, at baseline or after six months, there were no differences in antihypertensive medication prescription, and similarly no significant differences were observed on follow up after 6 months (Table 3). In addition, there were no differences at baseline or on follow up of pulse wave velocity, or pulse wave velocity derived parameters, augmentation indices, or diastolic relaxation index between the groups. Simple paired t testing showed significant increases in pulse wave velocity for the 1.0 mmol/l calcium dialysate (9.64 ± 1.94 vs 10.45 ± 1.98 m/s, p = 0.028) and 1.35 mmol/l calcium dialysate (9.75 ± 1.96 vs 10.21 ± 2.18 m/s, p = 0.02) groups, but not for the other two groups (Figure 1). Echocardiograms were available for review from 31 patients dialysing with calcium dialysate 1.0 mmol/l, 34 using 1.25 mmol/l, 106 using 1.35 mmol/l, and 16 dialysing with calcium ≥1.75 mmol/l. There were no differences in echocardiogram derived ejection fraction, left ventricular end diastolic diameter, intra-ventricular septal wall thickness, or left ventricular posterior wall thickness between the subsets of patients (Table 3).

**Table 3:** Blood pressure measurements and pulsewave velocity grouped according to dialysate calcium concentration

**Dialysate calcium**	**1.0 mmol/**	**1.25 mmol/l**	**1.35 mmol/l**	**≥1.5 mmol/l**
SBP1 mmHg	150.3 ± 28.8	144 ± 30.3	149.2 ± 28.1	150.8 ± 27.7
DBP1 mmHg	79.7 ± 14.1	76.6 ± 14.9	81.7 ± 16.3	80.8 ± 13.9
SBP2 mmHg	151.4 ± 22.5	147.6 ± 30.7	149.5 ± 29.5	148.5 ± 14.9
DBP2 mmHg	81.1 ± 14.6	79.8 ± 16.6	81.5 ± 18.1	77.3 ± 12.7
PP1 mmHg	70.5 ± 20.9	67.3 ± 22.8	67.6 ± 19.0	70.0 ± 20.8
PP2 mmHg	70.3 ± 16.2	67.8 ± 18.8	68.1 ± 18.8	69.3 ± 16.4
%Δ SBP	0(−11.4to15.2)	2.6(−5.9 to 41.9)	0 (−10.6 to 12.5)	−4.1 (−10.8 to 7.3)
%Δ DBP	2.5 (−12.1 to 16.1)	4.3 (−6.3 to 14.3)	−4.4 (−12.4 to 11.9)	−4.3 (−10.8 to 1.3)
PWV1 ms^-1^	9.64 ± 1.94	9.41 ± 2.17	9.75 ± 1.96	9.69 ± 2.13
PWV2 ms^-1^	10.45 ± 1.98	9.79 ± 2.25	10.21 ± 2.18	9.6 ± 2.11
%Δ PWV	5.6 (−3.2 to 17.8)	5.3 (−5.1 to 18.8)	2.6 (−10 to 21.8)	1.3 (−11.8 to 10.9)
SBPao1 mmHg	150.7 ± 32.1	144.7 ± 35.7	150.8 ± 33.2	153.4 ± 30.8
SBPao2 mmHg	153.0 ± 26.9	138.7 ± 32.9	150.9 ± 33.5	149.2 ± 30.6
AoAXi1 (%)	39.0 ± 16.6	37.5 ± 17.4	38.7 ± 16.2	41.4 ± 12.9
AoAXi2 (%)	40.4 ± 15.9	37.5 ± 14.2	40.3 ± 16.1	41.8 ± 14.4
BrAXi1 (%)	6.7 (−17.3 to 31.2)	−1.6 (−34.8 to 25.4)	4.1 (−29.7 to 31.2)	12.3 (−12.3 to 29.7)
BrAXi2 (%)	3.9 (−28.6 to 27.9)	−8.1 (−23.5 to 25.5)	4.3 (−20.2 to 25.5)	8.9 (−16.8 to 33.9)
DRA1	36.7 ± 15.8	44.7 ± 23.6	40.3 ± 16.6	38.1 ± 12.3
DRA2	42.1 ± 15.3	46.7 ± 21.1	43.5 ± 16.7	39.2 ± 16.2
Echo ej%	60(50–60)	60(55–60)	60(50–60)	60(50–60)
LVEDD cm	4.72 ± 0.75	4.65 ± 0.61	4.66 ± 0.73	4.70 ± 0.68
IVSD cm	1.27 ± 0.32	1.33 ± 0.25	1.28 ± 0.29	1.21 ± 0.24
PWD cm	1.28 ± 0.21	1.17 ± 0.24	1.18 ± 0.24	1.03 ± 0.18
ACEI/ARB (%)	25.9	37.3	27.9	19.1
β blocker (%)	24.1	25.4	27.3	38.1
CCB (%)	27.8	22	18.8	19.1
Hypo1 (%)	3.7	0	7.1	9.5
Hypo2 (%)	16.7*	3.3	7.1	0

Peripheral systolic blood pressure predialysis (SBP1) and after six months (SBP2), peripheral diastolic blood pressure (DBP), pulse pressure (PP), percentage change (%), aortic-femoral pulse wave velocity (PVW), aortic systolic blood pressure (AoSBP). Aortic augmentation index (AoAXi), brachial artery augmentation index (BrAXi), diastolic relaxation index (DRA) were corrected for heart rate. Cardiac echo derived left ventricular ejection fraction (ej), left ventricular end diastolic diameter (LVEDD), intra-ventricular septal thickness (IVSD), and left ventricular posterior wall thickness (PWD). Symptomatic hypotensive episodes during dialysis (Hypo). Results expressed as percentage (%), mean ± SD, or median (interquartile range). Patients dialysing with dialysate calcium 1.5 mmol/l (n = 17) added to those dialysing using calcium 1.75 mmol/l (n = 4) summated due to low numbers. * p > 0.05 anova with post hoc correction vs high calcium dialysate (≥1.5 mmol/l).

Recorded episodes of symptomatic intradialytic hypotension were greater on follow up for those dialysing against the lowest calcium dialysate, but there were no differences when episodes were averaged between the two pulse wave velocity measurements (Table 3).

## Discussion

Over the last decade there has been increased recognition of the clinical importance of vascular stiffness in dialysis patients due to both the high prevalence of hypertension in the dialysis patient with increased risk of stroke on one hand [15], and episodes of intradialytic hypotension [16], potentially compromising cardiac [17] and cerebral perfusion [18] on the other. Many studies have reported an association between vascular calcification and vascular stiffness in dialysis patients and increasing mortality [2]. In our cohort there was a small but statistically significant increase in pulse wave velocity over the six month period, but no change in blood pressure, augmentation index, or other derived parameters.

Previous small series comparing dialysate calcium concentrations of 1.0, 1.25 and 1.5 mmol/l, have suggested that using higher dialysate calcium concentration for a single haemodialysis session, can increase vascular stiffness measured by pulse wave velocity with a reduction in augmentation index [4]. This was supported by another study reporting increased pulse wave velocity using a 1.75 mmol/l dialysate calcium compared to 1.25 mmol/l [5]. The question arises as to whether these effects are simply due to temporary changes in calcium flux during dialysis affecting vascular reactivity, since higher dialysate calcium concentrations lead to a net influx of calcium during dialysis [19], and so reduce the incidence of intradialytic hypotension compared to lower concentrations [20]. In addition, other studies have reported changes in pulse wave velocity over six months when using higher dialysate calcium concentrations, both in peritoneal [10] and haemodialysis patients [8]. It is generally accepted that dialysate calcium concentrations of 1.25 mmol/l or less will usually lead to a net loss of calcium during a dialysis session, and those equal to greater than 1.5 mmol/l to a net gain [11], although it has been argued that even at 1.25 mmol/l there may theoretically be a calcium gain, and even calcium dialysate concentrations as low as 1.0 mmol/l may have neutral calcium balance [19]. Nevertheless, it is likely that repeated exposure to increased calcium gains may lead to increased microcrystal formation with resultant vascular calcification and aortic stiffness [20]. As such, we reviewed patients who had pulse wave velocity measurements six months apart using a device which has been validated against direct invasive measurements [12], and measuring the time between the first-direct and second-reflected wave, the time required for the wave to travel from the aortic root to the bifurcation and return and the distance travelled. This device does not overestimate pulse wave velocity as found with some carotid-femoral pulse wave velocity devices due to the effects of changes in velocity in muscular arteries.

Pulse wave velocity is well known to be affected by systolic hypertension, age, sex, body mass index and comorbid conditions including type 2 diabetes. As this was an audit of clinical practice, a range of dialysate calcium concentrations were used by differing numbers of patients. However despite this, our groups were well matched for those demographic factors which affect pulse wave velocity and baseline blood pressure. Unlike the previous reports, typically of 30 patients or fewer, we studied almost 290 patients, divided into 4 groups according to dialysate calcium concentrations. Whereas previous reports have observed increases in aortic stiffness comparing calcium dialysates of 1.0, 1.25 and 1.50 mmol/l [8]; 1.25 and 1.75 [5]; 1.5 and 1.75 [7]; and a reduction in pulse wave velocity following a reduction in dialysate calcium from 1.75 to 1.5 mmol/l [9], we were unable to demonstrate any differences between patients dialysing with calcium dialysate concentrations 1.0, 1.25, 1.35 and ≥1.5 mmol/l. There was a trend for pulse wave velocity to increase over time, which was statistically significant for those patients dialysing against a calcium dialysate of 1.0 mmol/l, and 1.35 mmol/l, but not the highest dialysate calcium concentrations. This may have simply been due to the relatively small number of patients dialysing with the highest dialysate calcium concentrations. Compared to previous reports the difference with our findings may have been due to the smaller numbers studied in earlier reports, coupled with selection bias and differences in pulse wave velocity measuring devices, particularly when the femoral artery was used. On the other hand our findings are in keeping with previous studies which observed no differences in central pulse pressure, carotid-radial pulse wave velocity, central or brachial augmentation indices [8]. Similarly others have not shown any change in aortic stiffness or pulse wave velocity with different dialysate calcium concentrations during a single dialysis session, with changes in blood pressure ascribed to changes in stroke volume [21].

As with other reports, patients dialysing against lower dialysate calcium had higher serum phosphate and parathyroid concentrations compared to those using higher dialysate calcium concentrations [22]. Although patients dialysing against the highest dialysate calcium concentrations were prescribed more calcium based phosphate binders, those dialysing against the lowest were prescribed more active vitamin D analogues, and more likely to be prescribed cinacalcet. We have no formal dietary information as to dietary calcium intakes, or assessment of patient compliance with prescribed phosphate binders and cinacalcet, although active vitamin D analogues were administered with dialysis sessions. As all patients were given the same standard dietary advice and dieticians working to achieve the same standards [23], it is likely that dietary calcium intake was similar, and certainly not greater in the lower dialysate calcium groups, such that any effect of dialysate calcium concentration would have been obscured by corresponding changes in dietary calcium. In addition as the group dialysing against the lowest dialysate calcium concentration were prescribed less calcium based phosphate binders, and more likely to be prescribed a non-calcium containing phosphate binder it supports our findings that the choice of dialysate calcium concentration does not affect pulse wave velocity in the short term.

In this study although there was a small but significant increase in pulse wave velocity for the whole cohort, we were unable to demonstrate any major difference in blood pressure, pulse wave velocity or derived indices according to calcium containing dialysates over a six month period. As our report is an audit of clinical practice, rather than a prospective trial, it was not designed to identify which factors were responsible for the increase observed. Although calcium containing microparticles are key to the initiation of soft tissue vascular calcification, the propensity for vascular calcification is somewhat more complex than simply reflecting changes in calcium influx and efflux during a dialysis session at the macro level. We were unable to demonstrate any differences in pulse wave velocity over 6 months associated with different dialysate calcium concentrations.

## Conclusions

Our findings however, do not imply that higher dialysate calcium concentrations cannot result in changes in vascular stiffness in the longer term as we investigated changes for only six months, and the differences in pulse wave velocity were small. Although our study may have been too short a period to demonstrate any measurable differences of different calcium dialysates, the increase in pulse wave velocity observed over time with the lowest calcium dialysate group does suggest that factors other than dialysate calcium may be more important in determining vascular stiffness in haemodialysis patients.

## Competing interests

The authors declare that they have no competing interests.

## Authors’ contributions

EC – measurements, data collection and writing manuscript AD – study design, analysis and writing manuscript. Both authors read and approved the final manuscript.

## Pre-publication history

The pre-publication history for this paper can be accessed here:

http://www.biomedcentral.com/1471-2369/14/189/prepub
